# Severe chronic periodontitis is not common in Acromegaly: Potential protective role of gingival BMP-2

**DOI:** 10.3906/sag-2006-93

**Published:** 2021-06-28

**Authors:** Sibel BAŞÇIL, Özlem TURHAN İYİDİR, Nilüfer BAYRAKTAR, Melek Eda ERTÖRER, Neslihan BAŞÇIL TÜTÜNCÜ

**Affiliations:** 1 Department of Periodontology, Faculty of Dentistry, Başkent University, Adana Turkey; 2 Department of Endocrinology and Metabolism, Faculty of Medicine, Başkent University, Ankara Turkey; 3 Department of Biochemistry,Faculty of Medicine, Başkent University, Ankara Turkey

**Keywords:** Acromegaly, periodontitis, growth hormone, bone morphogenetic protein-2, bone morphogenetic protein-4

## Abstract

**Background/aim:**

Advanced chronic periodontitis is observed rarely in acromegaly. Periodontal tissue including the alveolar bone is seemed to be spared from the systemic metabolic derangements of bone in this patient population. Chronic elevation of growth hormone, IGF-1, and bone morphogenetic proteins may play a role in periodontal tissue regeneration in acromegalics. In this study, we aimed to evaluate the potential roles of local gingival bone morphogenetic proteins (BMP) in periodontal tissue pathology in acromegaly.

**Materials and methods:**

Thirty-five patients with acromegaly and 22 healthy subjects were recruited. All the participants were examined by the same periodontologist for the diagnosis of periodontal diseases. BMP-2 and -4 were studied in gingival crevicular fluid.

**Results:**

Gingival BMP-2 and BMP-4 levels were similar in acromegaly and control groups in general, with and without chronic periodontitis. For all the participants, gingival BMP-2 levels were statistically lower in those participants with chronic periodontitis then those without periodontitis (29.4 ± 11.2 vs. 41.2 ± 23.2, respectively, p = 0.027). Causal relation between the gingival BMP levels and periodontal tissue health status was tested with one way ANOVA which revealed a significant difference between gingival BMP-2 levels in those with different degrees of periodontal tissue pathology (p = 0.025). When analyzed separately, gingival BMP-2 levels revealed a causal relation with the degree of periodontal pathology with borderline significance only in patients with acromegaly (p = 0.057).

**Conclusion:**

Acromegaly is a disease with an unexpectedly low frequency of advanced periodontitis, irrespective of the long disease duration and pathognomonic oral manifestations. BMP-2 might have a protective role against chronic advanced periodontitis in these patients.

## 1. Introduction: 

Acromegaly is among the rare diseases with complex effects on bone remodelling. Uncontrolled secretion of growth hormone (GH) from the hypophysis is the key feature of acromegaly, whereas elevated levels of insulin-like growth factor-1 (IGF-1), insulin-like growth factor binding protein-3 and calcitriol, and the presence or absence of secondary gonadal insufficiency are important factors that influence the bone metabolism in these patients (1). Duration of disease activity and remission are also important determinants of the final bone health (2).

Acromegaly has important pathognomonic oral features of prognathism, macroglossia, increased interdental spaces, and dental mobility (3). Although its typical characteristics are well known, periodonditis is unexpectedly rare, as demonstrated in some of the recent studies (4,5). Likewise in our previous study, we found that chronic periodontitis was not encountered more frequently, then the age and sex matched population and even surprisingly the advanced from of it was less commonly observed in acromegalic patients (6). 

Alveolar bone metabolism and thus, periodontal tissue seems to be spared or less affected by the complex influencing factors in acromegaly. Growth factors like GH, IGF-1, and GH-mediated local growth factors like bone morphogenetic proteins (BMPs) may play roles in periodontal tissue regeneration in this patient population. GH and IGF-1 are known to increase the formation of bone and hard tissues of the tooth (dentine, cementum, and enamel) (7). GH receptors are expressed in these tissues and can mediate local growth responses. There are few reports demonstrating the stimulation of bone morphogenetic protein-2 and -4 mRNA expression by GH and IGF-1 up to 4- to 5-fold in human dental pulp fibroblasts in vitro (8). BMP play an important role in periodontal tissue regeneration both in fetal life and in adults (9). The therapeutic use of recombinant BMPs like recombinant human (rh) BMP-2 has attracted considerable interest in the treatment of various periodontal diseases due to the potent ability of these factors to stimulate intramembranous bone formation without an endochondral intermediate (10).

In our present study, we hypothesized that the excess of GH/IGF-1 affects directly or indirectly the bone remodelling of the periodontal tissue in acromegaly patients. Long-standing GH/IGF-1 excess, by increasing the local tissue BMP levels, might influence positively the regenerative capacity of the periodontal tissue in this patient group. In this context, we aimed to determine BMP-2 and BMP-4 levels in the gingival fluid to find any association with the health status of the periodontal tissue in those with acromegaly.

## 2. Materials and methods

### 2.1. Study Subjects

We enrolled 35 acromegalic patients from the outpatient clinic of the Endocrinology Department at Başkent University Faculty of Medicine, consequently, between September 2010 and July 2013. The diagnosis of acromegaly was established clinically and confirmed by high serum GH concentrations that were not suppressed after oral glucose tolerance test (75 gr), elevated IGF-1 levels for age and sex, and demonstration of the pituitary tumor by computed tomographic scan or magnetic resonance imaging. Duration of the disease, history of pituitary surgery and radiotherapy, activity of the disease, treatment with a somatostatin analogue, and length of remission were recorded. A total of 22 otherwise healthy participants were enrolled for oral examination to assess periodontal tissue health and gingival BMP measurement. Exclusion criteria were history of chronic renal failure, hyperparathyroidism, hyperthyroidism, and active smoking, which may all influence bone metabolism and periodontal tissue. Informed written consent was obtained from all subjects prior to their enrollment, and our local ethics committee approved the study protocol (tracking number KA08/104).

### 2.2. Periodontal assesment 

Individuals from both groups were asked to fill a questionnaire about oral hygiene and frequency of annual check-up and/or regular dental visits.

All of the study subjects were examined by the same experienced periodontist. Periodontal pocket depth (presence of an abnormal gingival sulcus near the point at which the gums contact a tooth) was evaluated using periodontal probe at six different points of each tooth. Individuals with probing pocket depth (PPD) between 4-6 mm and clinical attachment level (CAL= amount of space between attached periodontal tissues and a fixed point, usually the cementoenamel junction) up to 4 mm were considered aschronic periodontitis with slight to moderate loss of periodontal support [15]. Those with PPD ≥ 6 mm and CAL greater than 4 mm were considered as chronic periodontitis with advanced loss of periodontal support (16). Bleeding on probing, which was considered an objective inflammatory parameter in periodontitis establishment, was measured using gingival bleeding index (GBI) (17). Gingival bleeding index ≤0.1 was considered as low risk for the development of periodontitis. Tooth mobility levels were evaluated and classified as degrees 1, 2, and 3. Radiographic examinations, tooth loss, diastemas (space or gap between two teeth), and malocclusion were also assessed.

### 2.3. Endocrinological assesment 

Clinical data of acromegalic patients were evaluated and assessed by the same experienced endocrinologist.

The estimated duration of the disease was determined according to the clinical history and review of the past photographs of the patients. The disease activity was assessed by GH suppression during OGTT. Hormonal status of anterior hypophysis was analysed by both basal serum pituitary hormone levels (ACTH, PRL, FSH, LH, TSH) with their end organ hormones and dynamic tests. Disease was considered cured when all these criteria were met: Random GH level less than 2.5 mg/L, plasma IGF-1 levels normal for the patient’s age, GH suppressed during OGTT, and the patient is not taking any somatostatine analog. Disease was defined as inactive when random GH level was less than 2.5 mg/L, and plasma IGF-1 levels were normal for the patient’s age under a somatostatin analog.

### 2.4. Biochemical assesment 

Serum levels of GH, IGF-1, and IGFBP-3 were determined by commercially available kits: chemiluminescent enzyme immunometric assay for GH (Immulite Growth Hormone, Diagnostic Products Corp., CA, USA), immunoradiometric assay (Diagnostic Systems Laboratories, DSL-6600 ACTIVE) for IGFBP-3, highly sensitive and specific immunoradiometric assay, which uses a modified version of the standard acid-ethanol extraction procedure (Diagnostic Systems Laboratories, DSL-5600 ACTIVE) for plasma IGF-1. 

The levels of BMP-2, and -4 in GCF samples were assayed using sandwich ELISA method (Aviscera Bioscience Inc., Santa Clara, CA, USA). All ELISA procedures were carried out according to the manufacturer’s instructions. Microcentrifuge tubes, containing a PerioPaper strip, with absorbed Gingival cevicular fluid (GCF) sample, were removed from storage and warmed to +4 °C, and then eluted using a centrifuge (11). The microcentrifuge tubes containing the strips and the dilution buffer were vortexed for 1 min and incubated overnight at +4 °C. The next morning, samples were vortexed for 3 min at room temperature. After the centrifugation at 1000 g 20 min, the strips were removed and the fluid was assayed by ELISA for BMP-2 and BMP-4. The ELISA plates were then assessed spectrophotometrically for their optical density (OD) at an absorbance of 450 nm. The levels of BMP-2 and BMP-4 in in each GCF sample were determined using the concentration values of the standards included in the kit. The detection limits were as follows; BMP2 < 46.87 pg/mL, BMP-4 < 3.9 pg/mL. The intra- and inter-assay variabilities were 4%–6% and 8%–12% for BMP-2 and BMP-4, respectively. 

Statistical analysis: Descriptive statistics for the study group are expressed as mean ± standard deviation for continuous variables and as proportions for ordinal (categoric) variables, such as sex, radiotherapy, presence of periodondititis, and malocclusion. Differences within patient clinical characteristics, BMP levels, and comparisons with the healthy control group were evaluated for statistical significance using the Student’s t-test and the Chi-square method where appropriate. Comparison of nonparametric groups (acromegaly disease duration) was analysed by using Mann–Whitney U test. Assumption of equal group variances for continuous variables were tested by Levene’s tets for Students t- test. To test the effect of gingival BMP levels on the severity of periodontal disease, one way ANOVA test was conducted. Assumption of equal group variances was tested by posthoc Bonferroni and Dunnett tests. Statistical significance was considered when a two-sided p value was 0.05 or below. For statistical analysis, Predictive Analytics Software (PASW-Release 18) statistical software was used.

## 3. Results:

A total of 35 acromegalic patients with a mean age of 46.7 ± 10.2 years, and 22 otherwise healthy control volunteers with a mean age of 42.4 ± 4.2 years were included in the study. All of the acromegalic patients underwent pituitary surgery and 15 patients received radiotherapy. Twenty-nine of the acromegalic patients were under somatostatin analog treatment. Twelve of the acromegalic patients had inactive or cured disease, 23 patients had active disease. There was only one patient whose disease was cured. 

The clinical characteristics and the gingival BMP levels of the study participants are given in Table 1. The acromegaly group and the healthy volunteers were matched with respect to age but the proportion of females was higher in the healthy control group (p = 0.034). About 45.7% of the acromegalic patients had pathognomonic acromegalic oral features like macroglossia, prognatism, and/or malocclusion. Diabetes or prediabetes was encountered in 11 of the acromegaly patients. Hypogonadism was diagnosed in 6 of the acromegalics, 4 of which were secondary and 2 were primary hypogonadism cases. Secondary hypothyroidism was present in 2 of the acromegalics, while there was no patient with primary hypothyroidism. Frequency of chronic periodontitis was higher in acromegalic patients (45.7% vs. 22.7%; p = 0.043). Although mild-to-moderate degree of chronic periodontitis was more frequent in acromegalics, advanced periodontitis cases were similar in number in both groups (5.7% vs. 4.5%; p > 0.05) (Table 1). In real life generally and in case of our present study, females have better oral hygiene (as noted from questionnaire about oral hygiene and frequency of annual check-up and/or regular dental visits in our study). Hence the predominance of females in the healthy control group have biased the results of our study. Male abundance and the frequent periodontitis in males in acromegalics (50%) increased the total cases with periodontitis in the acromegaly group in the present study. When the groups were matched with sex, the females and males in both groups of study participants revealed similar frequency of periodontitis (Table 1). There was also no increase in the frequency of any degree of periodontitis cases even in those with robust manifestations of pathognomonic oral features of acromegaly such as macroglossia, prognathism, and/or malocclusion. Gingival BMP-2 and BMP-4 levels were similar in the acromegaly and control groups (Table 1). 

**Table 1:** Clinical characteristics and the gingival BMP levels of the study participants.

	Acromegalypatients (n = 35)	Healthy controlgroup(n = 22)	p
Age (years)	46.7 ± 10.2	42.4 ± 4.2	NS*
Sex (F/M)	21/14	19/3	0.034**
Acromegaly disease duration	6.87 ± 3.2	-	-
Diabetes/prediabetes mellitus	11 (31.4%)	-	-
Primary or secondary hypogonadism	6 (17.1%)	-	
Secondary hypothyroidism	2 (5.7%)	-	-
Typical acromegalic oral featuresMacroglossia/prognathism/malocclusion	16 (45.7 %)	-	-
Chronic periodontitis (any degree) (n,%)	16 (45.7%)	5(22.7%)	0.043**
Advanced chronic periodontitis (n,%)	2 (5.7 %)	1 (4.5%)	NS**
Chronic periodontitis (any degree) in females (n,%)	9 (42.8%)	4 (21.9%)	NS**
Chronic periodontitis (any degree) in males (n,%)	7 (50%)	1 (33.3%)	NS**
Gingival BMP2 (pg/mL)	33.5 ± 20.4	39.2 ± 17.4	NS*
Gingival BMP4 (pg/mL)	36.4 ± 26.4	29.9 ± 14.6	NS*

NS = nonsignificant, BMP2: Bone mophogenetic protein-1; BMP4: Bone morphogenetic protein-4. *Student’s t test, ** Chi Square test

Plasma IGF-1, GH and IGFBP-3, and gingival BMP levels according to the disease activity in the acromegalic group of are given in Table 2. Both gingival BMP-2 and BMP-4 levels were similar in those with active and inactive acromegalics. Plasma GH, IGF-1, and IGFBP-3 levels were statistically high, as expected, in those acromegalics with active disease. There were 23 acromegalics with active GH secretion with a median disease duration of 4.5 years (interquartile range Q3-Q1 = 5.5 years). Median duration of acromegaly was 8 years (interquartile range Q3-Q1 = 4 years) in those with inactive disease. The duration of acromegaly was statistically longer in those with inactive disease (p = 0.01) (Table 2). Total duration of disease inactivity was minimum 6 months and maximum of about 3 years for these patients at the time of study enrollment. Disease duration and duration of disease inactivity did not correlate with the gingival BMP levels. 

**Table 2:** Gingival BMP levels according to disease activity of the acromegalic group.

	Acromegaly patientswith active disease(high GH/IGF-1 levels) (n = 23)	Acromegaly patientswith inactive disease(normal GH/IGF-1 levels) (n = 12)	p
GH (ng/mL)	7.4 ± 3.1	2.4 ± 0.2	0.003*
IGF-1 (ng/mL)	411.0 ± 129.5	245.9 ± 31.7	0.008*
IGFBP-3 (ng/mL)	4178.3 ± 1086.8	2056.0 ± 393.7	0.004*
Disease duration (years)	Median 4.5IQR: 5.5	Median :8IQR: 4	0.01+
Presence of diabetes/prediabetes	5/23 (21.7%)	4/12 (33.3%)	NS
Presence of hypogonadism	4/23 (17.4%)	2/12 (16.6.%)	NS
Presence of typical oral features(macroglossia/prognathism/malocclusion)	10/23 (43.4%)	6/12 (50%)	NS
Chronic periodontitis of any degree	10/23	6/12	NS
Gingival BMP2(pg/mL)	26.6 ± 14.7	40.3 ± 28.2	NS
Gingival BMP4(pg/mL)	37.7 ± 37.6	34.0 ± 14.7	NS

NS = nonsignificant BMP1: Bone mophogenetic protein 1 (pg/mL); BMP2: Bone morphogenetic protein 2 (pg/mL); GH: Growth hormone; IGF-1: Insulin like growth factor -1; IGFBP-3: Insulin like growth factor binding protein-3; IOR: Interquartile ratio (Q3-Q1), *Student’s t test, ** Chi Square test, + Mann–Whitney U test.

Frequency of chronic periodontitis and gingival BMP levels were similar in acromegalics regardless of disease activity at the time of study enrollment. Likewise, presence of diabetes or prediabetes, nor the hypogonadism influenced the periodontal tissue health in our patient population.

Table 3 gives the gingival BMP levels according to periodontal tissue health. When those with and without chronic periodontitis were subgrouped, gingival BMP-2 levels were statistically lower in those participants with chronic periodontitis then those without periodontitis (29.4 ± 11.2 vs. 41.2 ± 23.2, respectively, p = 0.027). Gingival BMP-4 levels were similar in those with and without periodontitis. 

**Table 3:** Gingival BMP levels in subgroups of study participants.

	Participants with chronic periodontitis (n = 21)	Participitants without periodontitis (n = 36)	p
Gingival BMP2 (pg/mL)	29.4 ± 11.2	41.2 ± 23.2	0.027*
Gingival BMP4 (pg/mL)	37.3 ± 5.8	30.9 ± 6.7	NS
Acromegaly group:	Acromegaly patients withchronic periodontitis (n = 16)	Acromegaly patients without chronic periodontitis (n = 19)	
Gingival BMP2 (pg/mL)	28.1 ± 11.6	39.9 ± 26.8	NS
Gingival BMP4 (pg/mL)	41.4 ± 34.2	31.86 ±17.9	NS
Control group:	Healthy controls with chronic periodontitis (n = 5)	Healthy controls withoutchronic periodontitis (n = 17)	
Gingival BMP2 (pg/mL)	33.5 ± 9.9	40.9 ± 9.9	NS
Gingival BMP4 (pg/mL)	24.1 ± 10.7	31.6 ± 15.4	NS

*Student’s t test.

When the acromegalics and healthy controls with and without chronic periodontitis were analysed separately, both the acromegalics and the healthy control group, revealed similar gingival BMP-2 and BMP-4 levels. 

Table 4 summarizes the relation of gingival BMP levels with the periodontal tissue health status in acromegalics and the control group. Causal relation between the gingival BMP levels and periodontal tissue health status was tested with one way ANOVA, which revealed a significant difference between gingival BMP-2 levels in those with different degrees of periodontal tissue pathology (p = 0.025). When analyzed separately, gingival BMP-2 levels revealed a causal relation with the degree of periodontal pathology with borderline significance only in patients with acromegaly (p = 0.057). As the gingival BMP2 levels decreased, periodontal pathology tended to advance to higher degrees in severity (Table 4 and Figure). No significant correlation existed between gingival BMP-4 level and periodontal tissue pathology in the acromegalics. Likewise, neither the gingival BMP-2 nor the BMP-4 levels had been found to be correlated with the periodontal pathology in the healthy controls (Table 4).

**Table 4:** One way ANOVA test results of the effect of gingival BMP2 levels on the severity of periodontitis in acromegaly patients and the healthy controls.

All participants (n = 57)				
	No periodontitis	Mild to moderate periodontits	Severe periodontitis	p
Mean Gingival BMP2 levels (pg/mL)	41.3 ± 22.2	29.3 ± 9.4	20.8 ± 8.2	0.025
Acromegaly patients (n = 35)				
Mean Gingival BMP2 levels (pg/mL)	41.7 ± 25.6	27.7 ± 8.6	17.7 ± 5.1	0.057
Healthy controls (n = 22)				
Mean Gingival BMP2 levels (pg/mL)	40.9 ± 18.9	33.5 ± 11.4	33.2	NS

NS: nonsignificant

**Figure:**
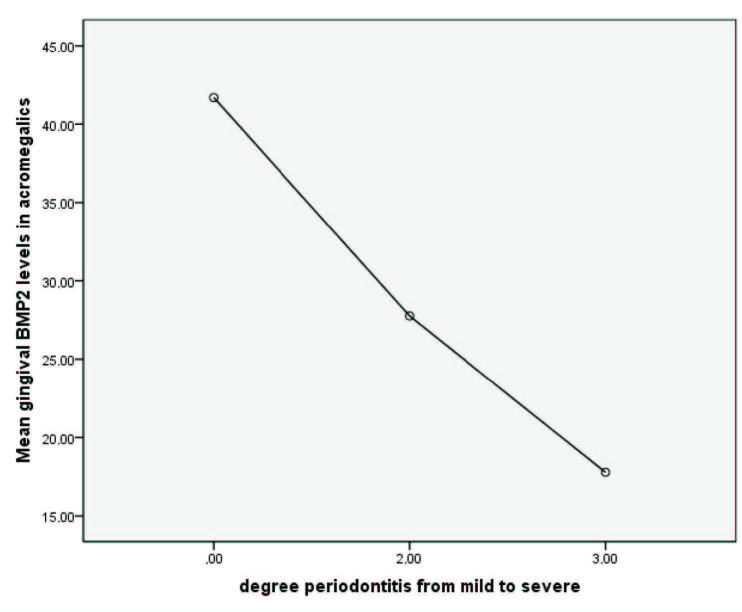
Mean BMP2 levels in groups of different periodontitis severity in the acromegalics. Degree of periodontitis: 0: No periodontitis; 1-2: Mild to moderate periodontits; 3: Severe periodontitis.

## 4. Discussion

In the previous studies exploring periodontal pathologies in acromegaly, periodontitis was found to be unexpectedly rare in these patients (6,12). In our present study, however, when both males and females were analysed together, periodontitis was found to be more frequent in the acromegaly group. Most probably, the female predominance in the control group might have biased the result. After matching the study participants with respect to sex, we found a similar frequency of chronic periodontitis in the acromegaly and the control groups. Although the typical oral manifestations like macroglossia, prognathism, and malocclusion were encountered in almost half of our acromegaly patients in the present study, the occurence, frequency, and grade of periodontitis were not increased. In accordance with this finding, the frequency of severe-advanced periodontitis was not increased in our acromegaly group and was similar to the control group. In a recent study of Ozdemir et al., lower occurrence of advanced periodontitis was encountered in the acromegaly patients (13). 

In our present study, we hypothesized that excess GH/IGF-1 affects bone remodelling in periodontal tissue via increasing the local BMP levels. This unexpected finding of the preservation of periodontal tissue in such a chronic disease with typical and pathognomonic derangements in oral tissue and anatomy can be explained by the long-term stimulation of local periodontal ligament cells with GH/IGF-1 and local increase of BMP family of proteins secondarily (8). In our study, although local BMP levels were similar in healthy and acromegalic participants, mean gingival BMP-2 levels differed according to the severity of periodontal tissue pathology. As the local gingival BMP2 levels increased, periodontitis severity decreased in the acromegaly patients. Such a relation with gingival BMP-2 levels was not seen in the healthy participants. This finding supports the suggested local protective and/or regenerative role of periodontal BMP-2 in acromegalic patients. We did not find a similar effect of BMP-4 levels on periodontal tissue health. Both BMP-2 and BMP-4 are the members of same family; however, they may have different effects on periodontal tissue (14). 

In our present study, although the gingival BMP2 levels tend to be higher in those with healthier periodontal tissue, gingival BMP-2 and BMP-4 levels were similar in the active and inactive acromegaly patients. Total acromegaly disease duration and duration of the inactive acromegaly period did not show any effect on the gingival BMP levels in the present study. However, this finding should be interpreted cautiously. Since acromegaly has a significant diagnostic delay in almost all patients (15), the duration of disease calculated from the time of biochemical diagnosis usually does not reflect exactly the actual cummulative exposure of tissues to the GH excess. Further prospective studies evaluating the periodontal status of patients with acromegaly may extend our knowledge on this issue. 

The relatively small sample size and the cross-sectional nature of our study are its major limitations. Considering this, and the fact that acromegaly is a very rare disease with a prevalence of 50–70 cases per million, it is usually hard to evaluate large numbers of cases in a single centre. We recruited the acromegaly patients and controls that we could reach from our acromegaly pool of hospital and get informed consent. We are also aware of the possible selection bias both in acromegalics and control subjects, which may not truly reflect the reality and thus, does not allow to make solid conclusions about the protective role of local gingival BMP levels in acromegaly patients. 

In conclusion, acromegaly is a disease with an unexpectedly low frequency of severe to advanced periodontal pathology, irrespective of the long disease duration and pathognomonic oral manifestations. Although we could not find any differences in local gingival BMP-2 and BMP-4 levels between patients in the acromegaly and control groups, the unexpectedly low frequency of severe periodontitis and the finding of increasing levels of gingival BMP-2 levels as the degree of periodontal tissue problem decreases in acromegalics, suggested a protective role for BMP-2 in acromegalics. As the gingival BMP2 levels decreased, periodontal pathology tended to advance to higher degrees in severity. To the best of our knowledge, this is the first study evaluating BMP-2 and -4 levels in the gingival fluid of patients with acromegaly. Our study is a pilot study and forms basis for further studies with larger sample sizes to evaluate the local regenerative and/or destructive processes and factors influencing the periodontal tissue biology in acromegaly patients.
